# Predicting osmotic potential from measurements of refractive index in cherries, grapes and plums

**DOI:** 10.1371/journal.pone.0207626

**Published:** 2018-11-16

**Authors:** Andreas Winkler, Moritz Knoche

**Affiliations:** Institute for Horticultural Production Systems, Leibniz-University Hannover, Hannover, Germany; Universita degli Studi di Perugia, ITALY

## Abstract

Studies of fruit tree water relations often require data on water potentials of fruit. However, this is sometimes difficult because the fruit stalks are not sufficiently long for use in a pressure bomb. Also, because fruit xylem function is often lost during maturation. In the absence of significant turgor, the osmotic potential of the expressed juice is a useful proxy for a fruit’s water potential. The osmotic potential of most fleshy fruit is determined largely by the concentration of soluble carbohydrates and this can be quantified by osmometry. Soluble solids may also be quantified by refractometry. Compared with osmometry, refractometry is markedly less expensive and also much faster. Hence, it is better suited to high-throughput analyses. The objective of this study was to establish relationships between the osmotic potentials of juices expressed from sweet cherries and sour cherries, grapes and plums as determined using a vapor pressure osmometer and their soluble solids concentrations as determined using a refractometer. The data reveal close relationships within all these species. Except for plums, the relationships between species were almost identical. This is due to similarity among cultivars and species in the relative abundances of the same set of major osmolytes—i.e. the carbohydrates glucose, fructose and sorbitol and the potassium salts of the organic acids malate or tartrate. For plums, the relationship between osmotic potential and soluble solids concentration was slightly displaced. Our findings indicate osmotic potentials may be reliably predicted from soluble solids concentrations determined by refractometry.

## Introduction

Information on fruit water potential is often needed in studies of tree water relations for example to characterize plant responses to soil water deficits [1]. However, fruit water potential is difficult to obtain. The most frequently-used technique, the Scholander pressure bomb, requires fruit to have sufficiently long stalks to fit through the gland in the bomb. Many fruitcrop species/cultivars do not satisfy this condition. A further requirement of the bomb method is that the xylem in the fruit and fruit stalk must be functional. This condition is also not met in many mature fruitcrop species (grape [2–10]; kiwi fruit [11,12]; cherries [13,14]; apple [15,16]; tomato [17,18]; mango [19]).

In most mature fruit, turgor is usually negligibly low (cherry [20,21], grape [22,23]), hence the osmotic potential of the fruit’s juice is a usefully proxy for the fruit’s water potential. Osmotic potentials are typically determined using an osmometer. The most common ones are freezing point osmometers and vapor pressure osmometers. The latter measure the dew point of the equilibrium atmosphere above a juice sample [24]. A freezing point osmometer requires samples to be free of suspended particles for accurate results [25]. However, when extracting juice from a fruit using a garlic press, this is typically not the case. To remove particles, a filtration or centrifugation step is usually necessary. Artefacts due to suspended particles are avoided with refractometry and also with vapor pressure osmometry. With a vapor pressure osmometer, the vapor pressure of the atmosphere in the sample chamber is quantified after it has reached equilibrium with the juice sample. Measurements require a stable temperature-controlled environment and a 1 to 2 min equilibration period per sample. Care must be taken to avoid sample-chamber contamination, so frequent and careful cleaning is required. Also, water vapor pressure osmometers must regularly be recalibrated against standard solutions. Together, these steps make vapor pressure osmometry quite time consuming and it also requires a clean, stable working environment.

Most of the osmolytes in the juice of a fleshy fruit are carbohydrates and these can be easily and accurately determined using a refractometer. In contrast to vapor pressure osmometers, refractometers are much more commonly available in plant science laboratories. There are even reliable handheld instruments designed for field use. Moreover, a refractometer is much less expensive than a vapor pressure osmometer (5 to 10% of the price but of similar accuracy), they are less time consuming and they are less demanding in their need for a clean, temperature-controlled environment. The temperature dependence of a refractometer results only from the temperature dependence of the refractive index of the solution under test (a known relationship which allows inbuilt electronic correction in a modern refractometer). For sugar solutions, the refractive index temperature dependence is much smaller than the vapor pressure temperature dependence.

The objective of this study was to determine relationships between osmotic potential, as determined by vapor pressure osmometry and soluble solids concentration, as determined by refractometry. We focused on juices expressed from sweet cherries, sour cherries, grapes and plums. So as to encompass as wide a range of osmolarities as possible, we used juice from developing and mature fruit. We also used juice from mature fruit that had been harvested and then subjected to conditions designed to raise their transpiration to a high level to further concentrate their soluble solids.

## Material and methods

### Plant materials

Fruit of the sweet cherry cultivars Adriana, Bing, Burlat, Dönissens Gelbe, Earlise, Early Korvic, Fabiola, Flamengo Srim, Gill Peck, Hedelfinger, Kordia, Merchant, NY242, Querfurter Königskirsche, Rainier, Regina, Sam, Samba, Schneiders Späte Knorpel, Staccato, Sweet Georgia and Sweetheart, of the sour cherry cultivars Achat, Morellenfeuer and Ungarische Traubige, of the grape cultivars Fanny, Nero and Riesling and of the plum cultivars Bergthold, Doppelte Hauszwetsche, Hauszwetsche Wolf, Jubilaeum, Mirabelle de Nancy, Toptaste and Wangenheim were sampled in three growing seasons. In total, we used 35 cultivars of four contrasting fruitcrop species. All fruit were harvested at the Horticultural Research Station of the Leibniz University in Ruthe, Germany (lat. 52°14’N, long. 9°49’E). Exceptions were the sweet cherry cultivars NY242, which was cultivated at the Esteburg research station, Jork, Germany (lat. 53°31′N, long. 98°40′E), and Bing and Sweet Georgia that were purchased locally, off-season. ‘Nero’ and ‘Fanny’ grapes were harvested at a commercial orchard in Gleidingen (lat. 52°16’N, long. 9°50’E). Sweet and sour cherry and grapes were used between the immature (transition stage II/III in cherries [26,27], veraison in grapes [28]) and the mature stage. Mass and color of the cherries ranged from a minimum of 4 g per fruit and yellow color (hue° 60) at the stage II/III transition to a maximum of 16 g per fruit and a dark red color (hue° 0) at maturity. For grapes, the respective data were 2 g per fruit and green color (hue° 110) at about veraison and a maximum of 7 g per fruit and red color (hue° 340) at the mature stage. For plums, the respective ranges were 10 g per fruit and red color (hue° 300 to 340) for the immature stage II and a maximum of 66 g per fruit and purple color (hue° 0 to 60) at maturity.

### Experiments

Fruit were first pitted (sweet and sour cherries and plums) and the juices were then expressed using a garlic press. Osmotic potentials and soluble solids concentrations of the juices were determined by vapor pressure osmometry (VAPRO 5520 and 5600; Wescor, Logan, UT) and by refractometry (DR6200-T; A. Kruess Optronic, Hamburg, Germany). An alternate expression of the soluble solids concentration (%) is the °brix, where 1°brix is defined as the refractive index of a 1% sucrose solution. To maximize the range in osmolarity, detached sweet and sour cherries and grapes were allowed to transpire above dry silica gel before extracting their juices. The number of individual fruit replicates was 1149 for sweet cherries, 125 for sour cherries, 157 for grapes and 69 for plums.

A separate experiment was conducted to study the effects of particles in juices obtained by expressing. This juice was prepared from Samba, Sweetheart and Regina sweet cherries as described above, transferred to 2 ml Eppendorf tubes (Eppendorf, Hamburg, Germany), and centrifuged at 20,800 *g*_n_ for 5 min. The osmotic potential and the soluble solids concentration of the juice before centrifugation, and those of the supernatant and the pellet after centrifugation were determined by vapor pressure osmometry (VAPRO 5520 and 5600; Wescor) and by refractometry (DR6200-T; A. Kruess Optronic). The osmotic potential of the pellet was read after 25 min of incubation. Preliminary experiments established that this time was necessary to equilibrate the pellet with the atmosphere in the sample chamber of the vapor pressure osmometer. The number of replicates was 44.

Relationships between the soluble solid concentrations or the osmolarity of solutions containing the pure substances of the major osmolytes of sweet and sour cherry, grape and plum were established. The major osmolytes were identified using literature sources [29]. They comprised glucose, fructose, sorbitol, malic acid and its potassium salt in sweet cherry and sour cherry, glucose, fructose and malic and tartaric acids and their potassium salts in grape and glucose, fructose, sorbitol, sucrose, malic acid and its potassium salt in plum.

### Statistical analyses

Data were analyzed by regression analysis using SigmaPlot (SigmaPlot 12.5, Systat Software, San Jose, CA) and R (Version 3.3.2; R Foundation for Statistical Computing, Vienna, Austria). Figures were created using SigmaPlot (SigmaPlot 12.5, Systat Software). Cubic polynomial regression models were fitted through plots of the osmotic potential of the juice (MPa) versus its soluble solids concentration (%).

## Results

**Fig 1:**
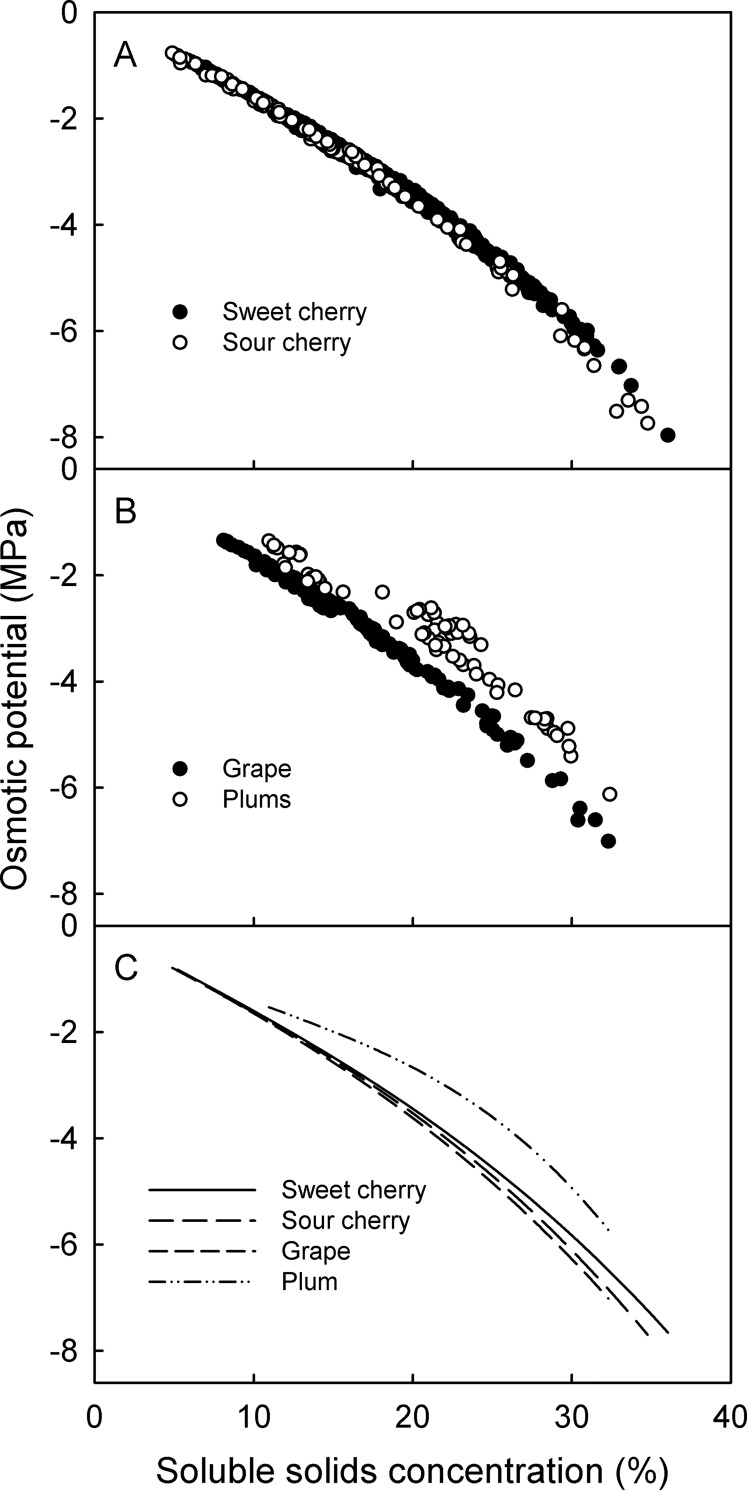
Relationship between soluble solids concentration and osmotic potential for juices of fleshy fruit. Juices were expressed from sweet and sour cherries (A) and grapes and plums (B). C) Regression lines redrawn and superimposed from the relationships depicted in A and B. Osmotic potentials were determined by vapor pressure osmometry, the soluble solids concentrations by refractometry. Data symbols in A and B represent individual fruit. For regression equations see Table 1.

Osmotic potentials of the juices of sweet and sour cherries, grapes and plums were all closely related to their soluble solids concentrations (Fig 1).

There were no differences in the relationships for juices from cherry fruit of different developmental stages, nor when mature fruit were allowed to transpire so as to concentrate their juices. Nor were there differences between cultivars but within species of sweet cherries, of sour cherries or of grapes. The sweet cherry cultivars investigated included fruit ranging in color from yellow (transparent juice) to dark red (highly colored juice). All juices followed the same relationship. Variations between species were only slightly larger than between cultivars but were surprisingly small (Fig 1). The exception was plum. Here, the relationship between osmotic potential and soluble solids concentration was slightly displaced compared to that of the other fruit species investigated. Also, variation in the relationships among the plum cultivars was somewhat larger compared with among sweet cherries or sour cherries or grapes (Fig 1C). The parameters of the regression equations and the coefficients of determination are given in Table 1.

**Table 1:** Range of osmotic potential, regression equations and the coefficient of determination for the relationships between osmotic potential (*π*) and the concentration of soluble solids (SSC) of sweet cherry, sour cherry, grapes and plums.

Species	Osmotic potential (MPa)		Equation	Coefficient of determination
	Min	Max		(r^2^)^a^
Sweet cherry	-0.8	-8.0	*π* (*MPa*) = 0.136 – 0.182 * SSC (%) + 0.002 * SSC^2^ (%) – 7.192 * 10^−5^ * SSC^3^ (%)	0.998
Sour cherry	-0.8	-9.6	*π* (*MPa*) = 0.123 – 0.186 * SSC (%) + 0.002 * SSC^2^ (%) – 9.08 * 10^−5^ * SSC^3^ (%)	0.998
Grapes	-1.3	-7.0	*π* (*MPa*) = 0.042 – 0.168 * SSC (%) + 5.294 * 10^−4^ * SSC^2^ (%) – 6.293 * 10^−5^ * SSC^3^ (%)	0.996
Sweet and sour cherry and grape^b^	-0.8	-9.6	*π* (*MPa*) = 0.239 – 0.207 * SSC (%) + 0.003 * SSC^2^ (%) – 1.088 * 10^−4^ * SSC^3^ (%)	0.996
Plums	-1.4	-6.1	*π* (*MPa*) = 1.412 – 0.413 * SSC (%) + 0.018 * SSC^2^ (%) – 3.797 * 10^−4^ * SSC^3^ (%)	0.966

^a^ The coefficient of determination of all equations are highly significant (<0.1%).

^b^ Because the cherries and grapes followed a practical identical relationship, we also provide an equation for the pooled data of these three species.

**Fig 2:**
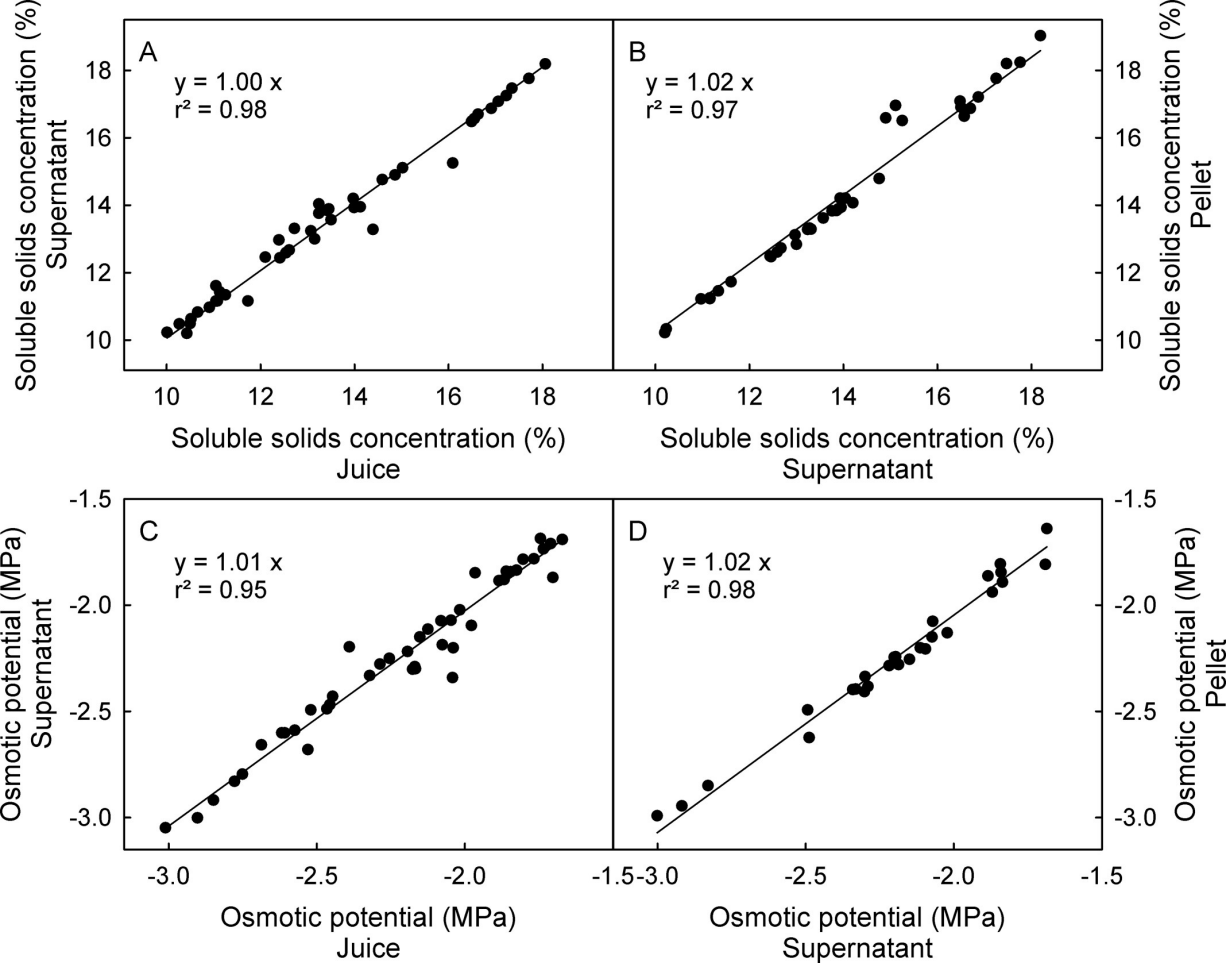
**Relationships between the soluble solids concentration (A-B) or the osmotic potential (C-D) with and without centrifugation**. Soluble solids concentration of sweet cherry juice without centrifugation vs. the supernatant (A) or pellet (B) of the same juice following centrifugation. Osmotic potentials of juice without centrifugation vs. the supernatant (C) or pellet (D) of the same juice following centrifugation. The juice was obtained by pressing pitted fruit. Osmotic potentials were determined by vapor pressure osmometry and the soluble solids concentration by refractometry.

The suspended particle content of the juice had no effect on osmotic potentials as determined by vapor pressure osmometry or on the soluble solids concentration as quantified by refractometry. There was no difference between juice that was centrifuged, regardless of whether the supernatant (particle free) or the pellet (slurry of particles) was analyzed compared to the particulate juice that was extracted without centrifugation. The slope of the regression equation between osmotic potential and soluble solid concentration did not differ significantly among all samples (Fig 2).

**Fig 3:**
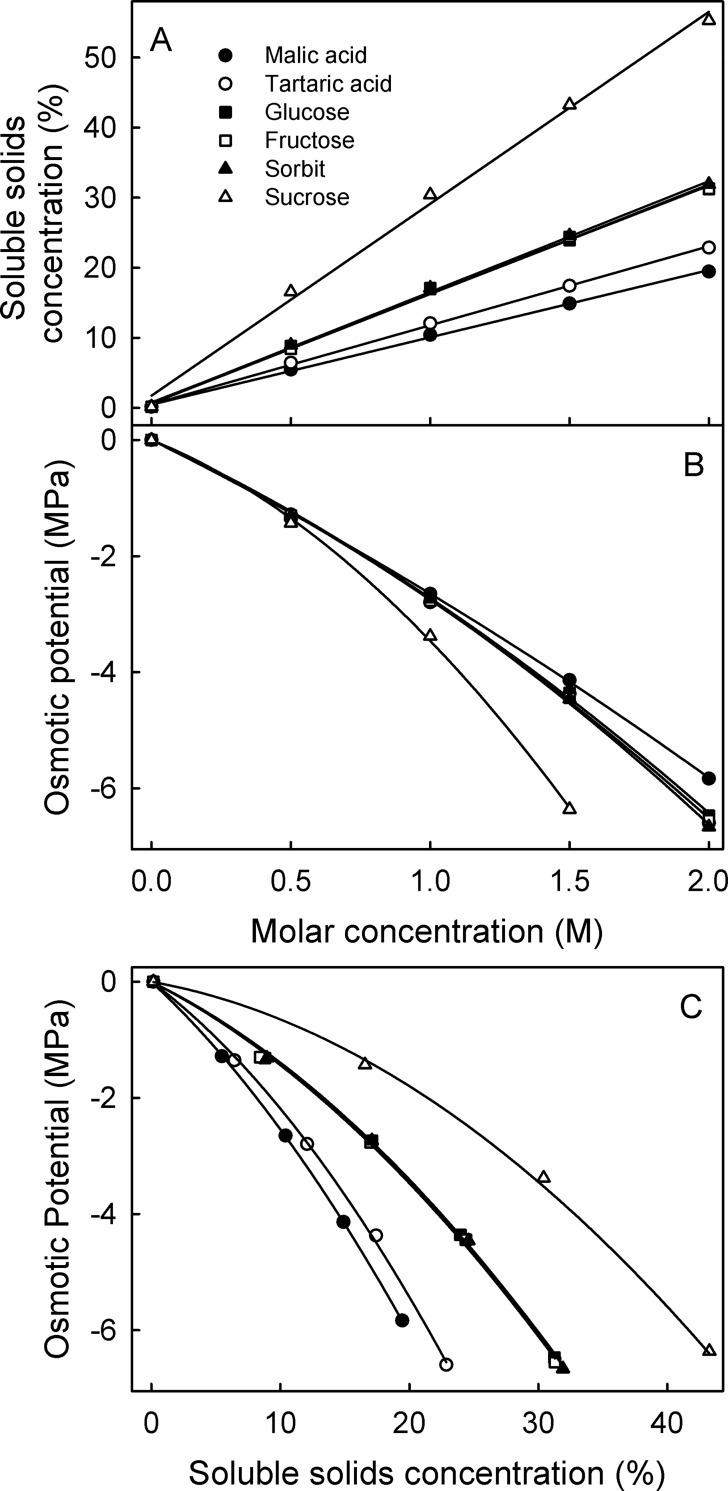
Relationships between the soluble solids concentration, osmotic potential and the molar concentration of pure osmolytes. The osmolytes selected represent the most abundant osmolytes in fleshy fruit. The 2 M sucrose solution was outside of the range of the vapor pressure osmometer.

Relationships between the soluble solids concentrations and the molar concentrations of pure substances of the most abundant osmolytes are linear. At any molarity, the concentration of sucrose was nearly twice that of glucose or of fructose or sorbitol–the latter three concentrations being essentially the same (Fig 3A). Also, malate and tartrate were similar but both were present in smaller amounts and, hence, lower concentrations compared to the monosaccharides at equal molarity.

Relationships between osmolarity and molar concentration of pure substances of the major osmolytes, however, were non-linear (Fig 3B). There was little difference between the different osmolytes. Only when the sucrose concentration exceeded 0.5 M, was the osmotic potential lower (i.e. more negative) than that of the other osmolytes (Fig 3B). At the same mass concentrations, the different osmolytes had different osmotic potentials. Osmolytes of similar molar mass had similar concentrations of soluble solids and thus similar osmotic potentials. As molar mass increased at a given soluble solids concentration, osmotic potentials increased (became less negative; Fig 3C).

## Discussion

The relationship between fruit juice osmotic potential and concentration depends on the soluble solids composition of the juice. This differs somewhat among species. The most abundant osmolytes in the juices of sweet and sour cherries and grapes are similar. Because the molar masses of these osmolytes are similar, their osmotic potentials and mass concentrations are similar and thus, the relationship between osmotic potentials and soluble solids are essentially identical. In sweet and sour cherry, 98.0% and 96.4% of total osmolarity are accounted for by the same five major osmolytes, i.e. glucose, fructose, sorbitol and malic acid and potassium malate (Table 2).

**Table 2:** Molar concentrations of abundant osmolytes in sweet and sour cherry, grape and plum^a^ at maturity.

Constituent		Molar concentration (mM)			
		Sweet cherry	Sour cherry	Grape	Plum
Organic acids	Malic acid	70.1 (6.7)^b^	161.1 (18.5)	33.7 (3.6)	91.0 (14.6)
	Citric acid	0.9 (0.1)	1.6 (0.2)	1.9 (0.2)	1.8 (0.3)
	Tartaric acid			27.6 (3.0)	
Sugars	Glucose	431.8 (41.1)	287.5 (33.1)	388.3 (41.9)	131.0 (21.0)
	Fructose	393.5 (37.5)	237.6 (27.3)	398.9 (43.0)	52.2 (8.4)
	Sorbitol	76.9 (7.3)	86.7 (10.0)	0.3 (0.0)	59.8 (9.6)
	Sucrose	4.4 (0.4)	12.3 (1.4)	12.1 (1.3)	215.0 (34.5)
Minerals	Potassium	56.3 (5.4)	65.2 (7.5)	51.2 (5.5)	58.8 (9.4)
	Calcium	4.2 (0.4)	4.0 (0.5)	3.2 (0.3)	3.2 (0.5)
	Magnesium	4.5 (0.4)	5.8 (0.7)	3.7 (0.4)	3.3 (0.5)
	Phosphate	6.5 (0.6)	7.1 (0.8)	5.8 (0.6)	6.5 (1.0)
Sum		1050 (100)	870 (100)	927 (100)	623 (100)

^a^ Molar concentrations are taken from [29].

^b^ The values in brackets represent the relative contributions (percentage) of the individual osmolytes to total osmolarity. The osmolarity was calculated assuming ideal behavior of the osmolytes and a 1:1 relationship between molarity and osmolarity.

Similarly, in grapes, 97.1% of the total osmolarity is accounted for by five osmolytes. In grape juice, in addition to four of the five most abundant osmolytes in sweet and sour cherry, tartaric acid and potassium tartrate contribute in significant amounts to the osmolarity. Due to the similarity in molar mass of these carbohydrates (range 180.2 to 182.2 g mol^-1^), and the comparatively lower concentrations of malate (134.1 g mol^-1^) and tartrate (150.1 g mol^-1^)–both having a slightly lower molar mass than the above carbohydrates—the similarity of the relationship is not surprising. Hence, we would expect fruit with similar compositions to follow the same relationships. Indeed, juice from mature blue berries (*Vaccinium corymbosum* L.), raspberries (*Rubus idaeus* L.), cape gooseberries (*Physalis peruviana* L.), gooseberries (*Ribes uva-crispa* L.) and red currants (*Ribes rubrum* L.) fit this relationship equally well (S1 Table). For juice from plums, however, the relationship was somewhat displaced. This displacement is likely due to the presence of sucrose as a major constituent. This has a much higher molar mass (342.3 g mol^-1^) and contributes about 34.5% of the total osmolarity of plum juice (Table 2). There is nearly no sucrose in sweet and sour cherries or in grapes. The other osmolytes are largely identical between sweet and sour cherry, grape and plum. That the displacement is due to sucrose is also consistent with the relationships between osmotic potential and soluble solids concentrations established for the pure substances. At the same mass concentration, sucrose solutions have a higher osmotic potential (less negative) than the others (Fig 3A). As a consequence, the relationship between soluble solids concentration and osmotic potential is different (Fig 3C). Andersen and Richardson [30] investigated the relationship between soluble solids concentration and osmotic potential of different osmolytes. The authors reported a positive relationship between molecular size of various osmolytes and the soluble solids concentration [30]. Peaches deviated from the relationship established in sweet cherries due to a different composition of osmolytes [30]. As in plum, sucrose is a major osmolyte in peach where it contributes 27.3% of total osmolarity [29]. Plotting our plum data in the same graph as the peach data of the literature [30] yielded relationships that were nearly superimposed.

## Conclusion

Refractometry is a robust method for estimating the osmotic potential of juice extracted from sweet and sour cherries, grapes and plums and, hence, for quantifying their respective water potentials. Refractometry is a rapid, reliable and inexpensive technique that avoids some of the shortcomings inherent to osmometry. The relationships established are new and valid for a wide range of osmolarities and cultivars within a given species. With the exception of plums, a single common relationship across a range of different species and cultivars is sufficient for most practical purposes.

## Supporting information

S1 Table Osmotic potentials and concentrations of soluble solids (SSC) of mature blue berries (*Vaccinium corymbosum* L.), raspberries (*Rubus idaeus* L.), cape gooseberries (*Physalis peruviana* L.), gooseberries (*Ribes uva-crispa* L.) and red currants (*Ribes rubrum* L.). The number of replicates (*n*) were 8 for blue berries, 38 for raspberries, 15 for cape gooseberries, 8 for gooseberries, and 37 for red currants. Data are means ± SE.
(DOCX)

S1 Dataset Raw data used in regression analyses. (XLSX)
