# Supplementary material for: Predicting osmotic potential from measurements of refractive index in cherries, grapes and plums
Source: PLoS One. 2018 Nov 16;13(11):e0207626. doi: 10.1371/journal.pone.0207626 (PMC6239309; doi:10.1371/journal.pone.0207626)
Supplement: S1 Table — The number of replicates (n) were 8 for blue berries, 38 for raspberries, 15 for cape gooseberries, 8 for gooseberries, and 37 for red currants. Data are means ± SE. (DOCX) [file pone.0207626.s001.docx]

**S1 Table. Osmotic potentials and concentrations of soluble solids (SSC) of mature blue berries (*Vaccinium corymbosum* L.), raspberries (*Rubus idaeus* L.), cape gooseberries (*Physalis peruviana* L.), gooseberries (*Ribes uva-crispa* L.) and red currants (*Ribes rubrum* L.).**

| Species | Osmotic potential (MPa) | Concentration of soluble solids (%) |
| --- | --- | --- |
| Blue berries | -2.3 ± 0.1 | 13.7 ± 0.5 |
| Raspberries | -1.5 ± 0.1 | 10.6 ± 0.3 |
| Cape Gooseberries | -1.9 ± 0.0 | 12.9 ± 0.3 |
| Gooseberries | -2.1 ± 0.0 | 13.9 ± 0.2 |
| Red currants | -2.1 ± 0.0 | 13.2 ± 0.2 |

The number of replicates (*n*) were 8 for blue berries, 38 for raspberries, 15 for cape gooseberries, 8 for gooseberries, and 37 for red currants.

Data are means ± SE.
